# COVID-19 in children across three Asian cosmopolitan regions

**DOI:** 10.1080/22221751.2020.1846462

**Published:** 2020-12-04

**Authors:** Gilbert T. Chua, Xiaoli Xiong, Eun Hwa Choi, Mi Seon Han, Sung Hee Chang, Byoung Lo Jin, Eun Joo Lee, Baek Nam Kim, Min Kyoung Kim, Kihyun Doo, Ju Hee Seo, Yae Jean Kim, Yeo Jin Kim, Ji Young Park, Sun Bok Suh, Hyunju Lee, Eun Young Cho, Dong Hyun Kim, Jong Min Kim, Hye Young Kim, Su Eun Park, Joon Kee Lee, Dae Sun Jo, Seung Man Cho, Jae Hong Choi, Kyo Jin Jo, Young June Choe, Ki Hwan Kim, Shuiqing Chi, Shao-tao Tang, Huan Qin, Li Shan Zhou, Peng Chen, Joshua Sung Chih Wong, Kate Ching Ching Chan, Felix Yat Sun Yau, Shu Yan Lam, Calvin Chit Kwong Chow, Tak Wai Wong, Victor Chi-man Chan, Grace Wing Kit Poon, Chun Bong Chow, Wilfred H. S. Wong, Yu Lung Lau, Godfrey Chi Fung Chan, Celine S. L. Chui, Xue Li, Marco Hok Kung Ho, Ian C. K. Wong, Paul Kwong Hang Tam, Kelvin K. W. To, Jong Hyun Kim, Patrick Ip, Mike Yat Wah Kwan

**Affiliations:** a Department of Paediatrics and Adolescent Medicine, The University of Hong Kong, Hong Kong, People’s Republic of China; b Department of Integrated Chinese and Western Medicine, Wuhan Children’s Hospital (Wuhan Maternal and Child Healthcare Hospital), Tongji Medical College, Huazhong University of Science and Technology, Wuhan, People’s Republic of China; c Department of Pediatrics, Seoul National University College of Medicine, Seoul, Korea; d Department of Pediatrics, Seoul Metropolitan Government-Seoul National University Boramae Medical Center, Seoul, Korea; e Department of Pediatrics, Seoul Medical Center, Seoul, Korea; f Department of Pediatrics, Hongseong Medical Center, Hongseong, Korea; g Department of Pediatrics, Seongnam Citizens Medical Center, Seongnam, Korea; h Department of Pediatrics, Gyeonggi Provincial Medical Center Ansung Hospital, Ansung, Korea; i Department of Pediatrics, Seonam Hospital, Seoul, Korea; j Department of Pediatrics, Gyeonggi Provincial Medical Center Icheon Hospital, Icheon, Korea; k Department of Pediatrics, Dankook University Hospital, Cheonan, Korea; l Department of Pediatrics, Samsung Medical Center, Sungkyunkwan University School of Medicine, Seoul, Korea; m Department of Pediatrics, Masan Medical Center, Changwon, Korea; n Department of Pediatrics, Chung-Ang University Hospital, Seoul, Korea; o Department of Pediatrics, Busan Medical Center, Busan, Korea; p Department of Pediatrics, Seoul National University Bundang Hospital, Seongnam, Korea; q Department of Pediatrics, Chungnam National University Hospital, Daejeon, Korea; r Department of Pediatrics, Inha University Hospital, Incheon, Korea; s Department of Pediatrics, Myongji Hospital, Goyang, Korea; t Department of Pediatrics, Pusan National University Hospital, Busan, Korea; u Department of Pediatrics, Pusan National University Children’s Hospital, Yangsan, Korea; v Department of Pediatrics, Chungbuk National University Hospital, Cheongju, Korea; w Department of Pediatrics, Jeonbuk National University Medical School, Jeonju, Korea; x Department of Pediatrics, Dongguk University College of Medicine, Gyeongju, Korea; y Department of Pediatrics, Jeju National University Hospital, Jeju, Korea; aa Department of Social and Preventive Medicine, Hallym University College of Medicine, Chuncheon, Korea; ab Department of Pediatrics, Incheon St. Mary’s Hospital, The Catholic University of Korea, Incheon, Korea; ac Department of Pediatric Surgery, Union Hospital, Tongji Medical College, Huazhong University of Science and Technology, Wuhan, People’s Republic of China; ad Institute of Maternal and Child Health, Wuhan Children’s Hospital (Wuhan Maternal and Child Healthcare Hospital), Tongji Medical College, Huazhong University of Science and Technology, Wuhan, People’s Republic of China; ae Department of Respiratory Medicine, Wuhan Children’s Hospital (Wuhan Maternal and Child Healthcare Hospital), Tongji Medical College, Huazhong University of Science and Technology, Wuhan, People’s Republic of China; af Department of Paediatrics and Adolescent Medicine, Princess Margaret Hospital, Hong Kong, People’s Republic of China; ag Department of Paediatrics, Faculty of Medicine, The Chinese University of Hong Kong, Hong Kong, People’s Republic of China; ah Department of Paediatrics, Queen Elizabeth Hospital, Hong Kong, People’s Republic of China; ai Department of Paediatrics and Adolescent Medicine, Tuen Mun Hospital, Hong Kong, People’s Republic of China; aj Department of Paediatrics and Adolescent Medicine, United Christian Hospital, Hong Kong, People’s Republic of China; ak Department of Paediatrics and Adolescent Medicine, Alice Ho Miu Ling Nethersole Hospital, Hong Kong, People’s Republic of China; al Department of Paediatrics and Adolescent Medicine, Pamela Youle Nethersole Eastern Hospital, Hong Kong, People’s Republic of China; am Department of Paediatrics and Adolescent Medicine, Queen Mary Hospital, Hong Kong, People’s Republic of China; an Centre for Safe Medication Practice and Research, Department of Pharmacology and Pharmacy, The University of Hong Kong, Hong Kong, People’s Republic of China; ao Department of Medicine, The University of Hong Kong, Hong Kong, People’s Republic of China; ap Research Department of Practice and Policy, UCL School of Pharmacy, University College, London, UK; aq Division of Paediatric Surgery, Department of Surgery, The University of Hong Kong, Hong Kong, People’s Republic of China; ar Dr. Li Dak Sum Research Centre, The University of Hong Kong-Karolinska, Institute Collaboration in Regenerative Medicine, The University of Hong Kong, Hong Kong, People’s Republic of China; as Department of Microbiology, Carol Yu Centre for Infection, Li Ka Shing Faculty of Medicine, The University of Hong Kong, Hong Kong, People’s Republic of China; at Department of Pediatrics, St. Vincent’s Hospital, The Catholic University of Korea, Suwon, Korea

**Keywords:** COVID-19, Asia, children, travel history, age-stratified

## Abstract

As another wave of COVID-19 outbreak has approached in July 2020, a larger scale COVID-19 pediatric Asian cohort summarizing the clinical observations is warranted. Children confirmed with COVID-19 infection from the Republic of Korea, the Hong Kong Special Administrative Region (HKSAR) and Wuhan, China, during their first waves of local outbreaks were included. Their clinical characteristics and the temporal sequences of the first waves of local paediatric outbreaks were compared. Four hundred and twenty three children with COVID-19 were analyzed. Wuhan had the earliest peak, followed by Korea and HKSAR. Compared with Korea and Wuhan, patients in HKSAR were significantly older (mean age: 12.9 vs. 10.8 vs. 6.6 years, *p* < 0.001, respectively) and had more imported cases (87.5% vs. 16.5% vs. 0%, *p* < 0.001, respectively). The imported cases were also older (13.4 vs. 7.6 years, *p* < 0.001). More cases in HKSAR were asymptomatic compared to Korea and Wuhan (45.5% vs. 22.0% vs. 20.9%, *p* < 0.001, respectively), and significantly more patients from Wuhan developed fever (40.6% vs. 29.7% vs. 21.6%, *p*=0.003, respectively). There were significantly less imported cases than domestic cases developing fever after adjusting for age and region of origin (*p* = 0.046). 5.4% to 10.8% of patients reported anosmia and ageusia. None developed pediatric multisystem inflammatory syndrome temporally associated with SARS-CoV-2 (PMIS-TS). In general, adolescents were more likely to be asymptomatic and less likely to develop fever, but required longer hospital stays. In conclusion, majority patients in this pediatric Asian cohort had a mild disease. None developed PIMS-TS. Their clinical characteristics were influenced by travel history and age.

## Introduction

The coronavirus disease 2019 (COVID-19) pandemic caused by the severe acute respiratory syndrome coronavirus 2 (SARS-CoV-2) was first reported in Wuhan, China in late December 2019. Subsequently, cases spread to other parts of China, then to neighbouring regions in Asia and worldwide. So far, more than 25 million people have been infected globally and over 860,000 have died due to COVID-19 [1]. The Republic of Korea (Korea) was one of the first Asian countries to be affected. The COVID-19 outbreak in Korea reached its peak in late February 2020, when a significant number of people were infected after attending a religious gathering [2]. In contrast, the COVID-19 outbreak in Hong Kong Special Administrative Region (HKSAR) appeared to peak in late March 2020 [3]. Most of the cases of COVID-19 in Hong Kong appeared to be imported from other parts of the world [4].

Studies on several regional pediatric cohorts from China and Europe have summarized the typical clinical characteristics of children with COVID-19 [5, 6]. However, a summary of the clinical observations in a large-scale Asian cohort of children with COVID-19 is still lacking. As another wave of COVID-19 outbreaks begin to sweep across the region in recent weeks, we could do well to learn from our clinical experiences in Asia during the first wave of COVID-19 to understand the differences between domestic and imported cases that can inform public health policy within the region.

## Objectives

In this study, we compared the clinical characteristics of children with confirmed COVID-19 during the first wave of local outbreaks in Korea, HKSAR, and Wuhan. We also compared the clinical characteristics between domestic and imported pediatric COVID-19 cases and between different age groups across the three Asian regions.

## Methods

### Study population

Children from Korea, HKSAR, and Wuhan below 19 years of age with confirmed COVID-19 by positive SARS-CoV-2 polymerase chain reaction (PCR) of a respiratory specimen were retrospectively included in the study. The three regions have similar case identification, admission and discharge policies. These patients were identified either because of having symptoms suspicious of COVID-19 infection, having epidemiological links to COVID-19 infected patients or returned from abroad. All patients were admitted to isolation facilities regardless of whether they were symptomatic or not. Children with COVID-19 infection in Korea were isolated at 20 hospitals and two non-hospital isolation facilities across the country. Children in Wuhan were all admitted to the Wuhan Children’s Hospital, which was the main center assigned by the central government for treating children diagnosed with SARS-CoV-2 infection in Wuhan. Children in HKSAR were admitted to eight paediatric units with airborne isolation facilities. Discharge criteria were being asymptomatic with two consecutive nasopharyngeal specimens negative for SARS-CoV-2 PCR at least 24 hours apart.

### Data collection

An electronic case report form was constructed in each region and merged for analysis. Physician-in-charge prospectively collected the clinical and laboratory data of each patient and entered into their regional centralized database, which was verified by the team leader. Information of these databases was combined for detailed analysis. The principal investigator would contact the corresponding doctors-in-charge for clarifications when necessary. Data including age at diagnosis, gender, travel history prior to COVID-19 confirmation, dates of admission and discharge and clinical symptoms were collected. The length of stay was defined as the duration between the dates of admission and discharge from the isolation facilities. All patients were classified into domestic and imported cases based on their travel histories. An imported case was defined by recent travel outside of the country within 2 weeks prior to symptoms onset. All patients were classified into different age categories: 0 to <1 year (infant), 1 to <5 years (pre-school), 5 to <12 years (primary school), and 12 to <19 years (secondary school).

### Ethics approval

This study was approved by the University of Hong Kong/Hospital Authority Hong Kong West Cluster Institutional Review Board (Reference number: UW 20-292) and the Research Ethics Board of the Wuhan Children’s Hospital (Reference number: WHCH 2020022). Ethics approval in Korea was obtained from the respective medical facilities as described previously [7].

### Statistical analysis

The statistical analyses were performed in SPSS version 26 (Armonk, New York, USA). Chi-square exact test was used to compare differences in the clinical symptoms and laboratory results between the Korean, HKSAR, and Wuhan cohorts, or between the different age groups. Fisher exact test was used when the conditions for Chi square were not met. One-way analysis of variances was used to detect differences in the mean age at diagnosis, mean length of hospital stay and laboratory results among the three Asian regions.

For the comparisons between domestic and imported cases, Chi square test was used to compare clinical symptoms, and unpaired *t*-test was used to compare mean age at diagnosis, mean length of stay and laboratory results. Multivariate regression analysis was used to adjust for potential confounders. Fisher exact test was used when the conditions for Chi-square were not met. The hospital admission in the Korean, HKSAR, and Wuhan cohorts were presented as plots by admission date. A two tailed *p*-value less than 0.05 was considered significant.

## Results

**Figure 1.**
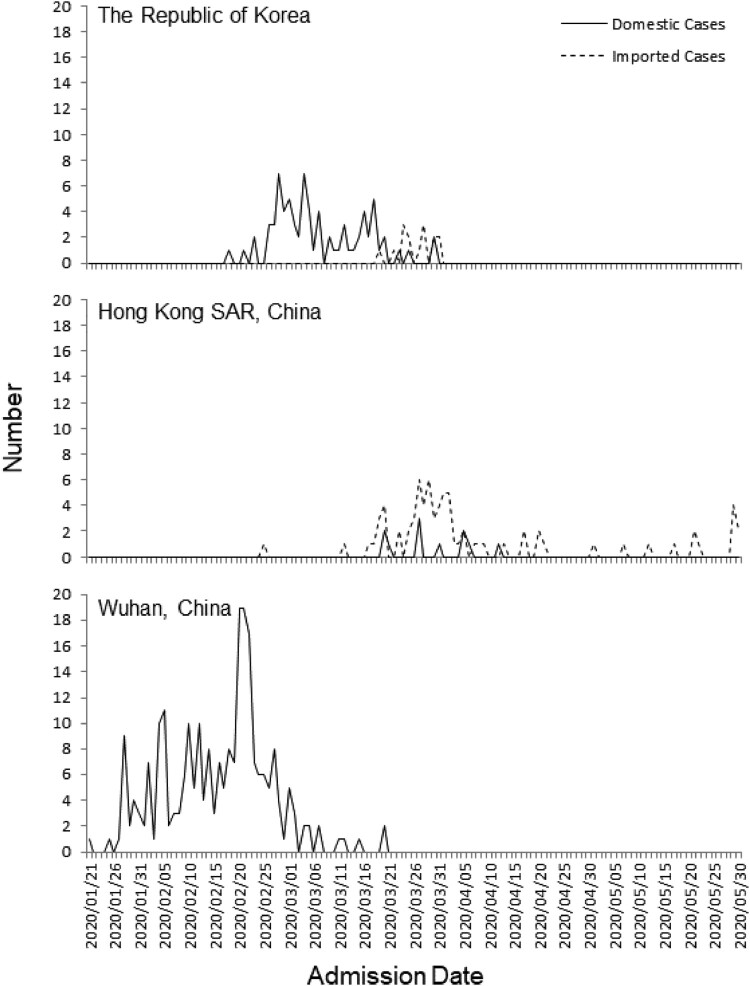
Distribution of the admission in domestic and imported pediatric COVID-19 cases in the Republic of Korea, and Hong Kong SAR and Wuhan during the first wave of COVID-19.

Figure 1 illustrates the distribution of pediatric COVID-19 cases between domestic and imported cases during the first wave in Korea, HKSAR, and Wuhan by admission date. Wuhan was first to reach a peak of COVID-19 pediatric cases in late February 2020, followed by Korea in mid-March, and HKSAR in late March to early April. The majority of the pediatric patients in HKSAR were imported cases, with admissions peaking in late March. The majority of the pediatric patients in Korea were domestic cases, and admissions peaked in late February to mid-March. In contrast, all pediatric COVID-19 cases in Wuhan were domestic cases. In Wuhan, the Wuhan Children’s Hospital was designated as the medical facility for treating children with COVID-19. All 244 children with COVID-19 admitted to this hospital between January 21 and March 20 were included. In Korea, the first pediatric COVID-19 patient was identified on February 18 [8]. From those admitted to 20 hospitals and two non-hospital isolation facilities up to March 31, 91 pediatric COVID-19 cases were included. In HKSAR, 88 pediatric COVID-19 cases admitted to eight pediatric hospitals between February 25 and May 31 were included.

Tables 1 and 2 show the comparison of the clinical characteristics and laboratory results of the 423 children with COVID-19 in the Korean, HKSAR, and Wuhan cohorts. Patients from HKSAR were significantly older than in Korea and Wuhan (12.9 vs. 10.8 vs. 6.6 years, *p* < 0.001, respectively). Significantly more patients from HKSAR had travelled outside of the region prior to COVID-19 confirmation compared to Korea and Wuhan (87.5% vs. 16.5% vs. 0%, *p* < 0.001, respectively). There were significantly more asymptomatic patients in HKSAR (45.5% vs. 22.0% vs. 20.9%, *p* < 0.001, respectively). More patients in Wuhan had a fever compared to Korea and HKSAR (40.6% vs. 29.7% vs. 21.6%, *p* = 0.003, respectively). Patients in Korea and HKSAR reported ageusia (10.8% and 6.8%, respectively) and anosmia (5.4% and 8.0%, respectively). There were some significant differences in the number of patients having cough, sputum, and abdominal pain. No patients developed rashes or signs of Kawasaki Disease or Pediatric Multisystem Inflammatory Syndrome Temporally associated with SARS-CoV-2 pandemic (PMIS-TS) compared to several cases reported in Western countries. No patients from Korea and HKSAR required intensive care support. One patient from Wuhan died of intussusception, which has been reported elsewhere [9].

**Table 1.** Comparison of the characteristics of pediatric COVID-19 cases in the Republic of Korea, Hong Kong SAR (HKSAR), and Wuhan.

	South Korea *N* = 91	HKSAR *N* = 88	Wuhan *N* = 244	*p*-value
^b^Age, mean ± SD (years)	10.8 ± 5.42	12.9 ± 5.5	6.6 ± 5.0	**<0.001**
^c^Male, *n* (%)	53 (58.2)	51 (58.0)	150 (61.5)	0.782
^c^Travel history, *n* (%)	15 (16.5)	77 (87.5)	0 (0)	**<0.001**
United Kingdom	4 (4.4%)	58 (65.9%)	/	
USA	8 (8.8%)	3 (3.4%)	/	
Pakistan	0 (0)	10 (11.4%)	/	
Spain	2 (2.2%)	1 (1.1%)	/	
Sweden	0 (0)	2 (2.3%)	/	
France	1 (1.1%)	1 (1.1%)	/	
Japan (Princess Diamond)	0 (0)	1 (1.1%)	/	
Singapore + Canada	0 (0)	1 (1.1%)	/	
^b^Length of hospital stay, mean ± SD (days)	17.3 ± 6.1	20.7 ± 11.6	12.6 ± 5.8	**<0.001**
Symptoms, *n* (%)				
^c^Asymptomatic	20 (22.0)	40 (45.5)	51 (20.9)	**<0.001**
^c^Fever	27 (29.7)	19 (21.6)	99 (40.6)	**0.003**
^c^Myalgia	7 (9.1)	3 (3.4)	9 (3.7)	0.120
^c^Rhinorrhea^a^	24 (26.7)	14 (15.9)	N/A	0.100
^c^Nasal congestion^a^	8 (8.9)	9 (0.2)	N/A	0.961
^c^Ageusia^a^	8 (10.8)	6 (6.8)	N/A	0.535
^c^Anosmia^a^	4 (5.4)	7 (8.0)	N/A	0.756
^c^Dizziness	1 (1.4)	0	3 (1.2)	0.568
^c^Cough	37 (41.1)	19 (21.6)	120 (49.2)	**0.003**
^c^Sputum	29 (32.2)	9 (10.2)	25 (10.2)	**<0.001**
^c^Diarrhea	11 (12.2)	8 (9.1)	15 (6.1)	0.179
^c^Abdominal pain^a^	6 (7.8)	2 (2.3)	4 (1.6)	**0.019**
^d^Vomiting^a^	6 (6.7)	1 (1.1)	N/A	0.118

Note: Bold values represent *p*-value <0.05.

^a^ Data from Wuhan was not available.

^b^ One-way analysis of variances was used.

^c^ Chi-square test was used

^c^ Fisher exact test was used.

**Table 2.** Comparison of the laboratory results of pediatric COVID-19 cases in the Republic of Korea, Hong Kong SAR (HKSAR), and Wuhan.

	South Korea Mean (SD)	Hong Kong Mean (SD)	Wuhan Mean (SD)	***p*-value**
Total White Cell (10^9^/L)	5.8 (1.7)	6.7 (3.2)	6.9 (2.6)	**0.004**
Haemoglobin (g/dL)	13.7 (1.3)	13.8 (1.4)	12.6 (1.5)	**<0.001**
Platelets (10^9^/L)	271.7 (95.0)	261.8 (83.0)	304.6 (100.6)	**0.001**
Neutrophils (10^9^/L)	2.6 (1.2)	3.6 (1.8)	3.3 (5.3)	0.325
Lymphocytes (10^9^/L)	2.5 (1.3)	2.4 (2.2)	3.3 (1.8)	**<0.001**
Prothrombin time (seconds)	12.4 (0.8)	12.1 (0.8)	11.1 (1.2)	**<0.001**
INR	1.1 (0.1)	1.1 (0.1)	0.9 (0.1)	**<0.001**
Activated partial thromboplastin time (Seconds)	32.3 (5.7)	30.7 (5.7)	33.3 (22.5)	0.814
Creatinine (micromol/L)	55.9 (20.4)	59.3 (17.8)	37.9 (22.7)	**<0.001**
Lactate dehydrogenase (U/L)	256 (109.8)	207 (59.6)	262.8 (100.0)	**<0.001**
Aspartate aminotransferase (U/L)	27.6 (10.4)	24.8 (4.5)	39.3 (53.0)	0.167
Alanine aminotransferase (U/L)	19.9 (12.6)	20 (9.7)	24 (41.0)	0.516
C-reactive protein (mg/dL)	0.2 (0.7)	1.9 (3.2)	3.7 (8.5)	**0.001**

Note: One-way analysis of variances was used. Bold values represent *p*-value <0.05.

Tables 3 and 4 show the in the clinical characteristics and laboratory results differences between domestic and imported cases. All patients from Wuhan were domestic cases. The majority of imported cases in HKSAR returned from the United Kingdom (65.9%) and Pakistan (11.4%); whereas imported cases in Korea returned from the USA (8.8%) and the United Kingdom (4.4%). Imported cases were significantly older than domestic cases (13.4 vs. 7.6 years, *p* < 0.001). More imported cases were asymptomatic (42.9% vs. 21.7%, *p* < 0.001), developed less fever (18.7% vs. 38.6%, *p* < 0.001) and cough (25.3% vs. 46.2%, *p* < 0.001), but required longer hospital stay (20.1 vs. 13.8 days, *p* < 0.001). Multivariate regression analysis showed that there was significantly less imported cases developed fever (*p* = 0.046) after adjusting for age and region of origin. There were no significant differences between imported and domestic cases in terms of lengths of stay, cough and being asymptomatic after the same adjustment.

**Table 3.** Comparison of the characteristics of domestic and imported pediatric COVID-19 cases from Republic of Korea, Hong Kong SAR (HKSAR), and Wuhan.

	Domestic cases *N* = 332	Imported cases *N* = 91	*p*-value
^b^Age, mean ± SD (years)	7.6 ± 5.3	13.4 ± 5.2	**<0.001**
^d^Male, *n* (%)	203 (61.1)	51 (56.0)	0.399
^b^Length of hospital stay, mean ± SD (days)	13.8 ± 6.6	20.1 ± 10.9	**<0.001**
Symptoms, *n* (%)			
^d^Asymptomatic	72 (21.7)	39 (42.9)	**<0.001**
^d^Fever	128 (38.6)	17 (18.7)	**<0.001**
^c^Myalgia	16 (5.0)	3 (3.4)	0.775
^d^Rhinorrhea^a^	21 (24.1)	17 (18.7)	0.481
^d^Nasal Congestion^a^	7 (8.0)	10 (11.0)	0.680
^d^Ageusia^a^	6 (8.1)	8 (9.1)	1.0
^d^Anosmia^a^	5 (6.8)	6 (6.8)	1.0
^c^Dizziness	3 (0.9)	1 (1.1)	1.0
^d^Cough	153 (46.2)	23 (25.3)	**<0.001**
^d^Sputum	46 (13.9)	17 (18.7)	0.333
^d^Diarrhea	24 (7.3)	10 (11.0)	0.346
^c^Abdominal pain^a^	9 (2.8)	3 (3.4)	0.726
^c^Vomiting^a^	6 (6.9)	1 (1.1)	0.06

Note: Bold values represent *p*-value <0.05.

^a^ Data from Wuhan was not available.

^b^ Unpaired *t*-test was used.

^c^ Fisher exact test was used.

^d^ Chi-square test was used

**Table 4.** Comparison of the laboratory results of domestic and imported pediatric COVID-19 cases from Republic of Korea, Hong Kong SAR (HKSAR), and Wuhan.

	Domestic cases Mean (SD)	Imported cases Mean (SD)	*p*-value
Total white cell (10^9^/L)	6.7 (2.4)	6.5 (3.1)	0.534
Haemoglobin (g/dL)	12.8 (1.5)	14.0 (1.2)	**<0.001**
Platelets (10^9^/L)	300.3 (99.2)	252.0 (83.4)	**<0.001**
Neutrophils (10^9^/L)	3.2 (4.8)	3.4 (1.8)	0.601
Lymphocytes (10^9^/L)	3.1 (1.7)	2.4 (2.1)	**0.002**
Prothrombin time (seconds)	11.1 (1.2)	12.2 (0.8)	**<0.001**
INR	0.9 (0.1)	1.1 (0.1)	**<0.001**
Activated partial thromboplastin time (seconds)	33.2 (21.8)	30.9 (5.9)	0.574
Creatinine (micromol/L)	41.0 (23.0)	61.2 (17.8)	**<0.001**
Lactate dehydrogenase (U/L)	261.6 (100.5)	212.6 (70.9)	**<0.001**
Aspartate aminotransferase (U/L)	37.2 (48.2)	25.4 (10.3)	0.304
Alanine aminotransferase (U/L)	23.3 (37.1)	19.4 (9.5)	0.358
C-Reactive Protein (mg/dL)	3.0 (7.7)	1.6 (3.1)	0.120

Note: Unpaired *t*-test was used. Bold values represent *p*-value <0.05.

Tables 5 and 6 show the differences in the clinical characteristics and laboratory results between different age groups. Significantly more children in the older age group had travel histories, were asymptomatic, and developed myalgia, nasal congestion, and sputum. On the other hand, significantly more children in the younger age group developed fever and cough.

**Table 5.** Comparison of the characteristics of children with COVID-19 by different age groups.

	0 to <1 *N* = 62	1 to <5 *N* = 67	5 to <12 *N* = 140	12 to <19 *N* = 154	*p*-value
^b^Male, *n* (%)	36 (58.1)	36 (53.7)	87 (62.1)	95 (61.7)	0.649
^b^Travel history, *n* (%)	4 (6.5)	7 (10.4)	14 (10.0)	66 (42.9)	**<0.001**
^c^Length of hospital stay, mean ± SD (days)	13.4 ± 7.8	13.9 ± 7.0	14.0 ± 7.1	16.6 ± 8.5	**0.012**
^b^Symptoms, *n* (%)					
Asymptomatic	4 (6.5)	13 (19.4)	43 (30.7)	51 (33.1)	**<0.001**
Fever	31 (50.0)	34 (50.7)	43 (30.7)	37 (24.0)	**<0.001**
Myalgia	0 (0.0)	2 (3.3)	4 (2.9)	13 (8.6)	**0.026**
Runny nose^a^	2 (25.0)	7 (33.3)	6 (13.3)	23 (22.1)	0.306
Stuffy nose^a^	2 (25.0)	2 (9.5)	0 (0.0)	13 (12.5)	**0.046**
Ageusia^a^	0 (0.0)	0 (0.0)	2 (4.5)	12 (11.7)	0.316
Anosmia^a^	0 (0.0)	0 (0.0)	1 (2.3)	10 (9.7)	0.273
Dizziness	0 (0.0)	0 (0.0)	2 (1.5)	2 (1.3)	0.647
Cough	43 (69.4)	30 (44.8)	50 (35.7)	53 (34.6)	**<0.001**
Sputum	11 (17.7)	5 (7.5)	16 (11.4)	31 (20.3)	**0.044**
Diarrhea	8 (12.9)	6 (9.0)	7 (5.0)	13 (8.5)	0.279
Abdominal pain^a^	0 (0.0)	0 (0.0)	7 (5.0)	5 (3.3)	0.127
Vomiting^a^	1 (12.5)	0 (0.0)	1 (2.2)	5 (4.8)	0.396

Note: Bold values represent *p*-value <0.05.

^a^ Data from Wuhan was not available.

^b^ Chi-square test was used

^c^ One-way analysis of variances was used.

**Table 6.** Comparison of the laboratory results of children with COVID-19 by different age groups.

	0 to <1 Mean (SD)	1 to <5 Mean (SD)	5 to <12 Mean (SD)	12 to <19 Mean (SD)	*p*-value
Total white cell (10^9^/L)	8.0 (3.1)	7.9 (3.4)	6.5 (2.2)	5.8 (1.6)	<0.001
Haemoglobin (g/dL)	11.6 (1.7)	12.3 (0.9)	13.0 (1.2)	14.0 (1.3)	<0.001
Platelets (10^9^/L)	374.6 (142.1)	299.1 (84.8)	282.5 (82.9)	257.6 (67.1)	<0.001
Neutrophils (10^9^/L)	2.4 (2.4)	4.1 (10.3)	3.3 (1.8)	3.2 (1.4)	0.216
Lymphocytes (10^9^/L)	4.8 (2.2)	4.3 (2.5)	2.5 (0.9)	2.0 (0.8)	<0.001
Prothrombin time (seconds)	11.2 (2.2)	11.1 (1.1)	11.1 (0.7)	11.5 (0.9)	0.220
INR	0.9 (0.1)	0.9 (0.1)	0.9 (0.1)	1.0 (0.1)	0.001
Activated partial thromboplastin time (seconds)	34.3 (9.2)	32.0 (6.6)	34.0 (33.8)	31.5 (4.8)	0.822
Creatinine (micromol/L)	26.5 (12.0)	35.5 (38.2)	40.6 (10.4)	61.7 (15.6)	<0.001
Lactate dehydrogenase (U/L)	316.8 (90.0)	310.3 (95.6)	232.7 (93)	206.7 (70.3)	<0.001
Aspartate aminotransferase (U/L)	57.3 (84.5)	39.3 (16.7)	34.9 (43.6)	22.9 (8.3)	<0.001
Alanine aminotransferase (U/L)	40.5 (74.7)	16.0 (8.2)	19.8 (19.1)	20.2 (13.1)	<0.001
C-reactive protein (mg/dL)	3.5 (6.1)	3.1 (9.8)	2.3 (6.1)	2.7 (6.7)	0.729

Note: One-way analysis of variances was used. Bold values represent *p*-value <0.05.

## Discussion

To the best of our knowledge, this is one of the first multi-regional Asian pediatric studies to summarize and compare the clinical characteristics of children infected with coronavirus during the first wave of the COVID-19 outbreak. We also explored the differences between imported and domestic cases and differences between age groups across the three regions.

First, almost all pediatric patients had a mild disease, 26% (111/423) of cases were asymptomatic, and only 34.2% (145/423) of patients developed a fever. A significant number of patients (6.8%– 0.8%) in our cohorts reported having anosmia and ageusia, which are unusual symptoms reported in other case series [10, 11]. There were no deaths, except one patient from Wuhan who died from intussusception, likely unrelated to COVID-19 [10, 11]. Younger children, especially those younger than 1 year old, developed fever, which was in line with other large-scale cohorts. Laboratory results were unremarkable. Although there were statistically significant differences in various laboratory results between groups, it is likely contributed by the age-related variation. A recent European study in April 2020 involving 582 children with COVID-19 showed that 8% of patients required intensive care, 4% required mechanical ventilation, and one patient required extracorporeal membrane oxygenation. Significantly more children younger than 12 months, being male gender, having pre-existing co-morbidities, and presenting with lower respiratory tract symptoms required intensive care [12]. Similarly, a study from China involving 2143 children with COVID-19 reported the majority of severe and critical cases occurred in children younger than 1 year [13]. Interestingly, none of the 423 children in our Asian cohort or the 2143 children in the study by Dong et al [13] developed Kawasaki Disease, despite it being normally more prevalent in Asia than in the west [14]. Similarly, none reported having Pediatric Inflammatory Multisystem Syndrome Temporally associated with SARS-COV-2 (PIMS-TS), which was reported in several case series from Europe and North America [15–18]. Whether East Asians are genetically non-susceptible to PIMS-TS needs to be investigated with larger-scale observation studies.

Second, the peak of the outbreaks differed in Korea, HKSAR, and Wuhan. Despite being closer to the epicenter of the initial outbreak, the majority of the pediatric cases in HKSAR were imported cases outside of China. The peak of these imported cases in late March occurred within the overall peak of COVID-19 cases in HKSAR [3], which also coincided with the beginning of the outbreak in Europe and with the World Health Organization declaring a pandemic on March 11, 2020 [19]. We found the imported COVID-19 cases were significantly older than the domestic cases. Many were teenage students studying abroad in Europe or North America, who chose to return home as schools and universities closed during the COVID-19 pandemic. In contrast, the peak of COVID-19 outbreaks in Korea and Wuhan were much earlier [13, 20]. The domestic pediatric cases in Korea and Wuhan were significantly younger and had no travel histories, and were therefore more likely to have been from infections in the community.

Third, there were significant differences in the clinical characteristics between imported and domestic cases and between different age groups. More imported cases and older children were asymptomatic and fewer developed fever or respiratory symptoms. Compared to Wuhan, which were all younger domestic cases, HKSAR had the most imported cases and older children with COVID-19. Although all patients were managed under the same admission and discharge criteria, and no paediatric patients in our cohort breached the discharge criteria due to tight bed demands, patients required significantly longer hospital stay in HKSAR than in Wuhan (20.7 ± 11.6 days vs. 12.6 ± 5.8 days, *p* < 0.001). Furthermore, our analysis demonstrated that travel history remained to be a significant factor associated with the development of fever, after adjustment for age and region of origin. We postulate that this might be related to imported cases of different SARS-CoV-2 strains from different parts of the world in various phases of the pandemic. A recent study showed there was a shift from the initial predominant D614 strain in China to the G614 strain that was dominant in Europe, North America, and Oceana, which eventually found its way to Asia (including China and HKSAR). This study suggested that the G614 strain is associated with a higher viral load, but not an increase in disease severity [21]. Both strains were shown to be effectively neutralized by SARS-CoV-2 neutralizing antibodies in convalescent plasma [22]. An in-vitro study also demonstrated that the G614 strain is capable of transmitting more efficiently than the D614 strain, but G614 does not bind more efficiently to the angiotensin-converting enzyme 2 (ACE2), the receptor for SARS-CoV and SARS-CoV-2 [23]. Bunyavanich et al. demonstrated there was an age-dependent expression of ACE2 in the nasal epithelium and ACE2 gene expression was lowest in young children, which might explain why children with COVID-19 have less severe illness than adults [24]. Nevertheless, whether the G614 strain is associated with a more insidious infection needs to be further investigated.

Given that 26% of children with COVID-19 in our Asian cohort were asymptomatic, only a third developed a fever, and imported cases were less likely to develop fever, public health measures reducing the risk of community spread need to be continued. Simple measures that appeared to be useful during the SARS epidemic such as temperature screening [25] may not be sufficient for the COVID-19 pandemic. Compared to the high number of asymptomatic COVID-19 cases, the majority of SARS cases presented with fever, but serological study showed asymptomatic SARS carriers were extremely rare [26, 27]. Nevertheless, other measures such the universal use of face masks, border controls, and mandatory self-quarantine policies for travellers returning from high-risk areas should be kept in place until an effective COVID-19 vaccine becomes available [28].

This study needs to be interpreted with the following caveats. First, not all strains of SARS-CoV-2 have been identified. However, the imported cases from March 2020 onwards were predominantly associated with the G614 strain, and epidemiological studies confirmed a shift in the dominant strain in the community. Second, Korean pediatric COVID-19 cases included in this study comprised 14.5% (91/627) of cases up to March 31, 2020 [29], whereas almost all pediatric cases from HKSAR and Wuhan were included in the analysis. Nevertheless, the Korean patients were identified from 22 isolation facilities in different parts of Korea, and the clinical characteristics should be representative of pediatric patients during the first wave of COVID-19 in Korea. Third, specific data, such as gestational age, of affected infants were not included in this study, which may affect the interpretation of their clinical pictures. Finally, pediatric data from European countries and North America were not available for analysis in this study. Future collaborative studies will be needed for a direct comparison of the clinical characteristics of pediatric COVID-19 cases between different ethnicities.

In conclusion, the majority of pediatric COVID-19 cases in Asia were mild and a significant proportion was asymptomatic. Kawasaki disease or PIMS-TS was not seen in our COVID-19 cohort. Compared to domestic cases, significantly more patients returning from overseas were asymptomatic and these imported cases were less symptomatic. Further studies are needed to investigate if the less symptomatic and asymptomatic imported cases are associated with newer strains, and whether this requires further measures to avoid future waves.
